# Crystal structure of domain of unknown function 507 (DUF507) reveals a new protein fold

**DOI:** 10.1038/s41598-023-40558-y

**Published:** 2023-08-18

**Authors:** Cole E. McKay, Jianlin Cheng, John J. Tanner

**Affiliations:** 1 Department of Biochemistry, University of Missouri, Columbia, MO 65211 USA; 2 Electrical Engineering and Computer Science Department, University of Missouri, Columbia, MO 65211 USA; 3 Department of Chemistry, University of Missouri, Columbia, MO 65211 USA

**Keywords:** SAXS, X-ray crystallography

## Abstract

The crystal structure of the domain of unknown function family 507 protein from *Aquifex aeolicus* is reported (AaDUF507, UniProt O67633, 183 residues). The structure was determined in two space groups (*C*222_1_ and *P*3_2_21) at 1.9 Å resolution. The phase problem was solved by molecular replacement using an AlphaFold model as the search model. AaDUF507 is a Y-shaped α-helical protein consisting of an anti-parallel 4-helix bundle base and two helical arms that extend 30-Å from the base. The two crystal structures differ by a 25° rigid body rotation of the C-terminal arm. The tertiary structure exhibits pseudo-twofold symmetry. The structural symmetry mirrors internal sequence similarity: residues 11–57 and 102–148 are 30% identical and 53% similar with an E-value of 0.002. In one of the structures, electron density for an unknown ligand, consistent with nicotinamide or similar molecule, may indicate a functional site. Docking calculations suggest potential ligand binding hot spots in the region between the helical arms. Structure-based query of the Protein Data Bank revealed no other protein with a similar tertiary structure, leading us to propose that AaDUF507 represents a new protein fold.

## Introduction

The interrelationship among amino acid sequence, three-dimensional structure, and protein function is a fundamental pillar of biology. The sequence-structure–function principle enables the classification of proteins into groups with shared characteristics and the annotation of possible functions. The utility of the sequence-structure–function principle is especially notable in the post-genomic era where the number of known protein sequences far exceeds the capacity of experimental characterization.

Nearly 40% of known proteins lack any annotation within public databases and are usually referred to as hypothetical proteins^1^. These proteins lack sufficient sequence similarity to proteins of known function to apply sequence-structure–function relationships. Hypothetical proteins may be grouped into families, such as domains of unknown function (DUFs). DUFs are a large set of families within the Pfam database^2^ (now part of InterPro database^3^) that do not include any protein of known function^4^. DUFs constitute an appreciable fraction of the protein family universe. Pfam 26.0 includes 3526 DUF families, corresponding to 26% of all the Pfam-A (curated) families^2^.

Goodacre et al. conceived the idea of “essential” DUFs as a way to prioritize DUFs for experimental and computational characterization^5^. By intersecting the Database of Essential Genes with the Pfam DUF database, they generated a list of over 300 essential proteins in 16 model bacterial species containing 238 DUFs, most of which represent single-domain proteins. Here we have studied an essential DUF from the DUF507 family. DUF507 corresponds to Pfam family PF04368, which has been integrated to InterPro family IPR007463. The family comprises approximately 3000 proteins from 231 proteomes. DUF507 proteins have two architectures, having either ~ 90 or ~ 180 residues.

Herein we report two crystal structures of the DUF507 family protein from the hyperthermophilic bacterium *Aquifex aeolicus* (AaDUF507, UniProt O67633, 183 residues). The structure of AaDUF507 reveals a pseudo-twofold symmetric Y-shaped protein consisting of a 4-helix bundle base and two 30-Å helical arms. In one of the structures, electron density for an unknown ligand may indicate a functional site. AaDUF507 appears to represent a new protein fold.

## Results

### Secondary and tertiary structure of AaDUF507

**Figure 1:**
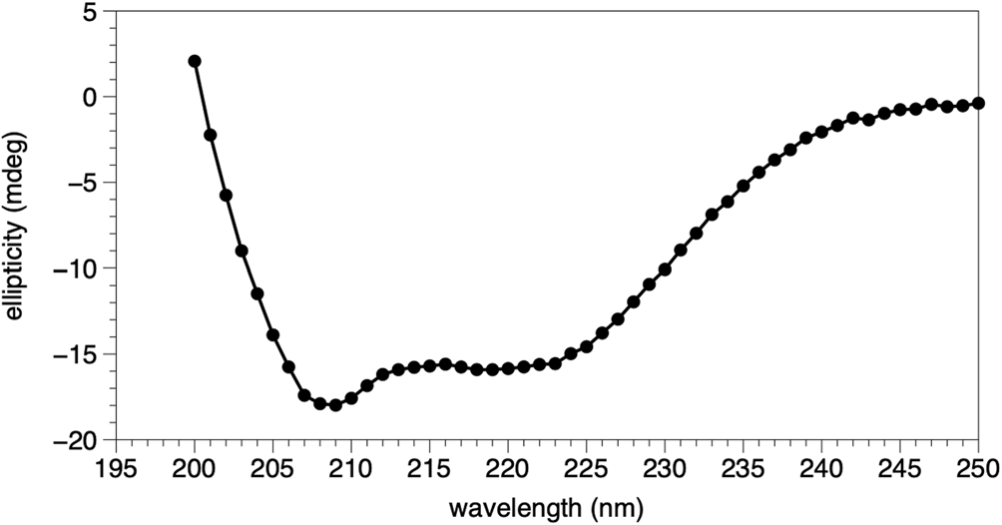
Far-UV circular dichroism spectra of AaDUF507 collected at 24 °C.

Circular dichroism (CD) was used to study the secondary structure of AfDUF507 in solution. The far UV CD spectrum exhibited features near 222 and 208 nm, characteristic of proteins with high α-helical content^6^ (Fig. 1).

The crystal structure of AaDUF507 was determined at 1.9 Å resolution in two different space groups (*C*222_1_ and *P*3_2_21) (Table 1). The phase problem was solved with molecular replacement using a model from AlphaFold^7^ as the search model. Use of the AlphaFold model was motivated by the lack of detectable sequence similarity of AaDUF507 to any protein in the Protein Data Bank (PDB^8,9^). The final refined 2*F*_o_ − *F*_c_ electron density was strong throughout the entire chain, allowing all 183 residues to be modeled in both structures.

**Table 1:** X-ray diffraction data processing and refinement statistics.

Space group	*C*222_1_	*P*3_2_21
Unit cell parameters (Å)	*a* = 72.71 *b* = 86.53 *c* = 79.14	*a* = 83.10 *c* = 68.80
Resolution (Å)	45.5–1.87 (1.91–1.87)	41.5–1.90 (1.94–1.90)
Wavelength (Å)	1.0722	1.0722
*R*_merge_(*I*)^a^	0.081 (0.618)	0.079 (1.611)
*R*_meas_(*I*)^a^	0.088 (0.668)	0.084 (1.741)
*R*_pim_(*I*)^a^	0.034 (0.253)	0.026 (0.643)
Observations^a^	144,119 (8975)	221,069 (9359)
Unique reflections^a^	20,928 (1311)	21,999 (1365)
Mean I/σ^a^	14.9 (3.2)	18.6 (1.1)
CC_1/2_^a^	0.998 (0.879)	0.999 (0.575)
Completeness (%)^a^	99.8 (99.0)	99.8 (98.5)
Multiplicity^a^	6.9 (6.8)	10.0 (6.9)
No. of atoms		
Protein	1510	1498
Water	91	55
*R*_cryst_^a^	0.217 (0.385)	0.205 (0.305)
*R*_free_^a,b^	0.253 (0.413)	0.224 (0.343)
rmsd bonds (Å)	0.003	0.004
rmsd angles (°)	0.468	0.634
Ramachandran plot^c^		
Favored (%)	98.90	99.45
Outliers (%)	0.00	0.00
Clashscore^c^	2.58 (100th)	2.61 (99th)
MolProbity score^c^	1.25 (99th)	1.35 (98th)
Average *B* (Å^2^)		
Protein	37.3	40.4
Water	39.3	44.7
Coord. error (Å)^d^	0.26	0.20
PDB code	8T8K	8T8L

^a^Values for the outer resolution shell of data are given in parenthesis.

^b^5% test set.

^c^From MolProbity. The percentile ranks for Clashscore and MolProbity score are listed in parenthesis.

^d^Maximum likelihood-based coordinate error estimate from PHENIX.

**Figure 2:**
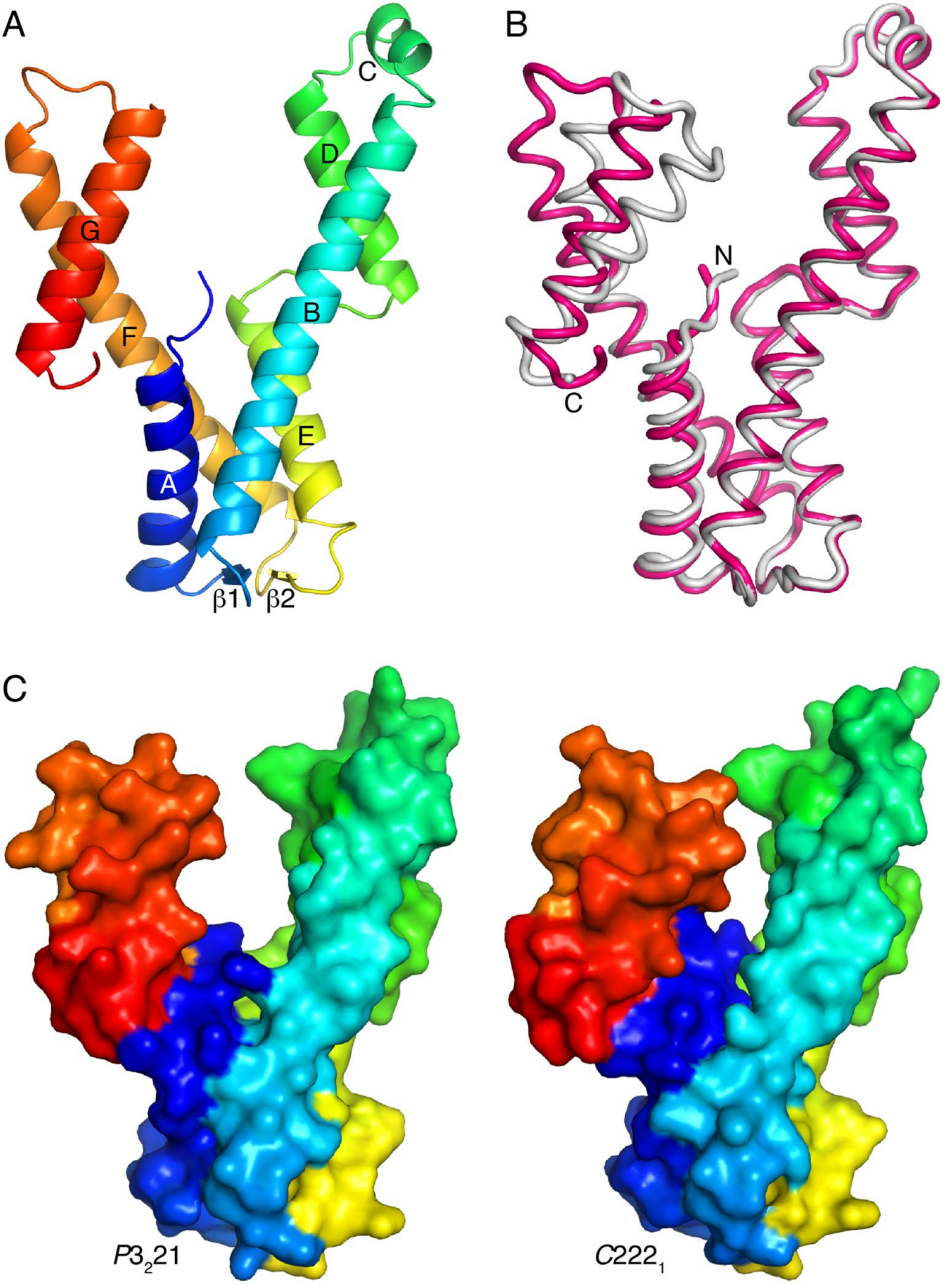
Fold of AaDUF507. (**A**) Cartoon representation of AaDUF507 (*P*3_2_21 structure). The chain is colored in a rainbow scheme with blue at the N-terminus and red at the C-terminus. Secondary structure elements are labeled (letters for α-helices, numbers for β-strands). (**B**) Superposition of the *P*3_2_21 (hot pink) and *C*222_1_ (white) structures. (**C**) Surface representations of the *P*3_2_21 and *C*222_1_ structures.

AaDUF507 is a Y-shaped predominantly α-helical protein (Fig. 2). The base of the Y consists of an up–down–up–down antiparallel 4-helix bundle formed by α-helices αA and αE, plus the N-terminal halves of αB and αF (Fig. 2A). A short, anti-parallel 2-stranded β-sheet connects the two halves of the bundle. The arms of the Y are connected to the base by the long αB and αF helices, which belong to both the base and the arms. The N-terminal arm consists of the C-terminal half of αB, plus αC and αD. The C-terminal arm consists of the C-terminal half of αF, plus αG.

The two crystal structures reveal the same fold and differ mainly in the angle of the C-terminal arm (Fig. 2B). The RMSD between the two structures is 2.4 Å for Cα atoms, calculated after superposition of all Cα atoms in CNS^10^. Most of this rather large deviation is contributed by the C-terminal arm (residues 141–183). For example, with the superposition based on all Cα atoms, the RMSD for residues 1–140 is only 1.2 Å, while that of residues 141–183 is 4.3 Å. The structural differences in the C-terminal domain may be described as 25° rigid body rotation, as determined with DynDom^11^. Because of the rigid body rotation, the arms are farther apart in the *P*3_2_21 structure than in the* C*222_1_ structure (Fig. 2C).

**Figure 3:**
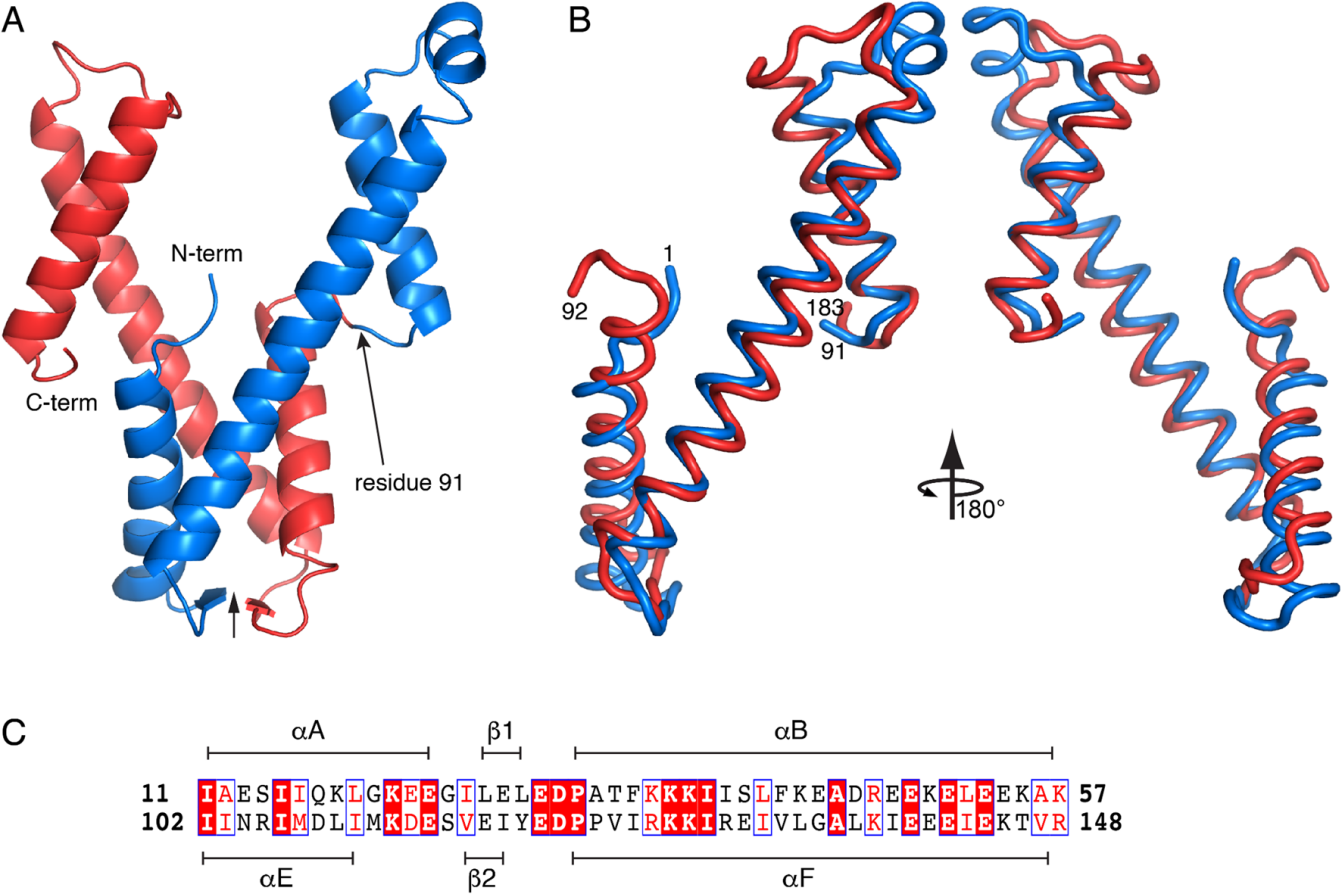
Pseudo-twofold symmetry of AaDUF507, as observed in the *P*3_2_21 structure. (**A**) Cartoon representation showing the location of the pseudo-twofold axis (vertical arrow) and the splitting of the protein into structurally similar N-terminal (blue) and C-terminal (red) halves at residue 91. (**B**) Superposition of the C-terminal half of AaDUF507 (red) onto the N-terminal half (blue). (**C**) Local sequence alignment of the two halves of AaDUF507 (30% identity, 53% similarity, E-value = 0.002).

The tertiary structure of AaDUF507 exhibits pseudo-twofold symmetry. This aspect of the structure is most apparent for the *P*3_2_21 structure (Fig. 3). The pseudo-twofold axis runs between the two β-strands of the base of the Y, and the protein may be split into two structurally similar halves corresponding to residues 1–91 and 92–183 (Fig. 3A). Structure alignment calculations with PDBeFold^12^ show that the N- and C-terminal halves align with a Q-score of 0.47 and RMSD of 1.98 Å over 75 aligned residues (Fig. 3B). The two halves of the fold differ mainly in the connecting segments at the tips of the arms, between αB and αD in the N-terminal half, and between αF and αG in the C-terminal half. These two connectors differ in secondary structure, with αB and αD linked by a helix (αC), and αF and αG connected by a loop. The two halves of the protein are also similar in sequence. Pairwise sequence alignment performed with BLASTP^13^ using the default options shows that residues 11–57 and 102–148 are 30% identical and 53% similar with an E-value of 0.002 (Fig. 3C). This region of the structure corresponds to αA and αB of the N-terminal half, and αE and αF of the C-terminal half, along with the β-sheet (Fig. 3C).

### Oligomeric structure of AaDUF507

**Figure 4:**
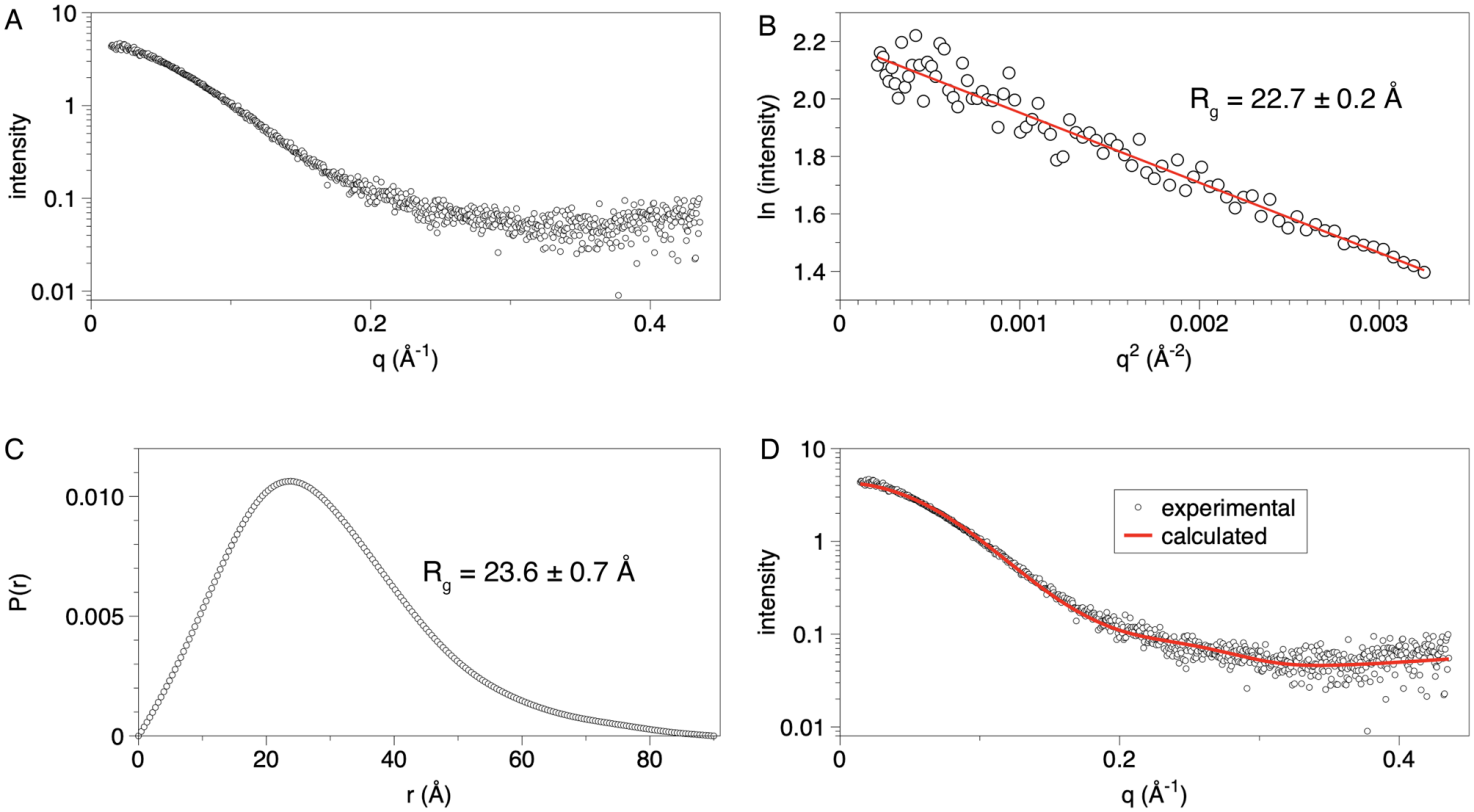
SAXS analysis of AaDUF507. (**A**) SAXS curve measured at 1 mg/mL. (**B**) Guinier plot. (**C**) Distance distribution function. (**D**) Comparison of the experimental SAXS curve (symbols) with the curve calculated from the crystal structure model (*P*3_2_21 structure). The goodness-of-fit parameter from FoXS (χ^2^) is 0.13.

The oligomeric structure of AaDUF507 was studied with small-angle X-ray scattering (SAXS). SAXS data were measured at several protein concentrations between 1 mg/mL and 16 mg/mL. Visual inspection of the scattering curves and Guinier analysis suggested the presence of nonspecific aggregation at protein concentrations above 1 mg/mL. Therefore, only the data set collected at 1 mg/mL was kept for analysis (Fig. 4A). Guinier analysis yields a radius of gyration (*R*_g_) of 23 Å (Fig. 4B, Table 2). Calculations of the distance distribution function suggest an *R*_g_ of 24 Å and maximum particle dimension of ~ 90 Å (Fig. 4C). For reference, the *R*_g_ of the crystal structure is 20 Å. These results suggest that the protein is not substantially self-associated in solution at 1 mg/mL. Furthermore, the molecular weight estimated from SAXS is 20.0–22.6 kDa, which is within 3–9% of the predicted molecular weight of 21.9 kDa for the monomer (Table 2). These results suggest that AaDUF507 is mainly monomeric at 1 mg/mL.

**Table 2:** Solution structural properties of AaDUF507 from SAXS.

Guinier analysis	
* qR*_g_ range	0.33–1.30
* R*_g_ (Å)	22.7 ± 0.2
*P*(*r*) analysis	
Points used	1–665
* D*_max_ (Å)	90
* R*_g_ (Å)	23.6 ± 0.7
Porod Vol. (Å^3^)	39,065
MW (kDa)	
Bayesian Inf.^a^	20.6 (− 6%)
MoW^a^	20.0 (− 9%)
* V*_c_^a^	22.6 (+ 3%)
SASBDB	SASDSX6

^a^The percent difference from the theoretical MW of the monomer (21.9 kDa) is listed in parentheses.

The SAXS data were further analyzed by calculating theoretical SAXS data from the crystal structure using FoXS^14,15^. The curves calculated from the two structures show good agreement with the experimental curve, as characterized by FoXS χ^2^ values of 0.13 and 0.17 for the trigonal and *C*-orthorhombic structures, respectively (Fig. 4D).

The crystal structures also imply a monomeric protein. Analysis of the protein–protein interfaces in the two crystal structures using PDBePISA^16^ did not reveal any assemblies predicted to be stable in solution. In summary, considering the SAXS data, which reports on the oligomeric state at low protein concentration, and the crystal structures, which provide information on oligomeric structure at high protein concentration, we conclude that AaDUF507 is predominantly monomeric in solution.

### AaDUF507 represents a new protein fold

The structure of AaDUF507 was compared to those in the PDB to uncover potential relationships to functionally characterized proteins and explore the possibility that it represents a new fold. Searching the PDB using TopSearch^17^, PDBeFold and DALI^18^ with either crystal structure as the query failed to return a global match. The top hits covered less than 60% of the query structure and displayed diverse functionalities. Some of the hits matched the 4-helix bundle base, while others recognized non-contiguous helices in larger proteins such as integral membrane proteins and dynein heavy chain. More meaningful hits were obtained when the 4-helix bundle was used as the query, as expected for a fold that is highly represented in the PDB^19^. The best of these hits had 80–90% query coverage and RMSD of 2.4–2.6 Å and displayed diverse functionalities (e.g., phenylalanine ammonia-lyase, PDB 1Y2M; α-domain of Transcription factor TFIIA, PDB 5M4S). These results suggest that AaDUF507 represents a new protein fold containing a 4-helix bundle domain.

### Adventitious ligand bound to AaDUF507

**Figure 5:**
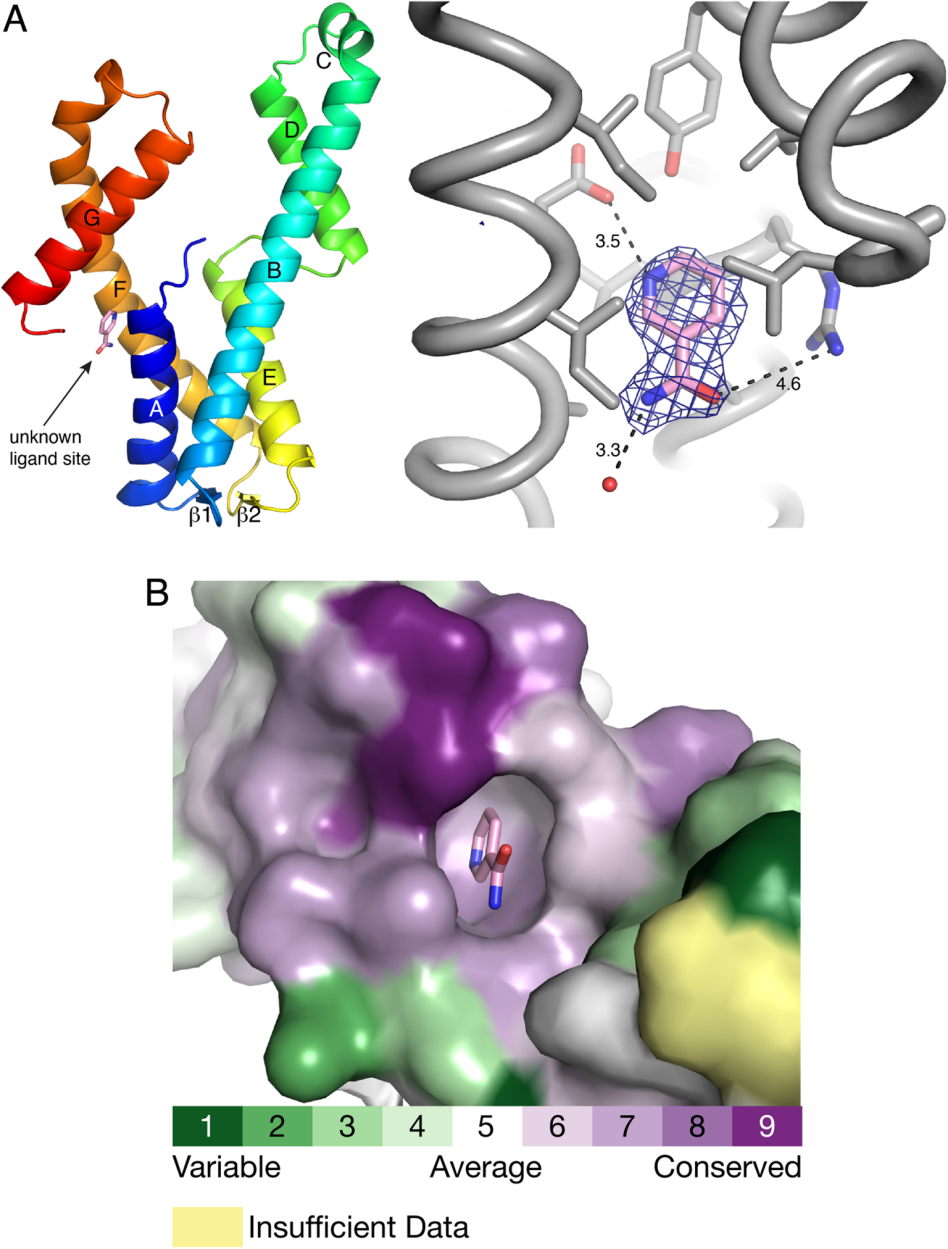
Unknown ligand site in the *C*222_1_ structure. (**A**) Location of the ligand site (left) and electron density (*F*_o_ − *F*_c_, 3σ) (right). For illustration purposes, the ligand has been modeled as nicotinamide. (**B**) Surface representation of the binding pocket. The surface is colored according to sequence conservation calculated with ConSurf.

The electron density map for the *C*222_1_ structure indicated an unknown ligand bound to the protein. The electron density resembled a 6-membered aromatic ring with a small, planar functional group attached (Fig. 5A). This molecular topology does not match any of the components of the protein buffer or crystallization solution. The density could be modeled satisfactorily as nicotinamide, although its true identity is unknown. Nicotinamide seems plausible because of the good fit to the electron density, and as a molecule of NAD metabolism, it presumably is present in the *Escherichia coli* cell cultures used to express the recombinant protein^20^. Trial refinement of nicotinamide with occupancy of 1.0 resulted in a mean atomic displacement parameter of 39.2 Å^2^, which is comparable to the mean *B*-factor of the protein of 37.3 Å^2^. Nevertheless, nicotinamide was not included in the final coordinates deposited in the PDB.

The unknown ligand site is a pocket located in the junction between the 4-helix bundle and the C-terminal arm, near the intersection of αA, αF, and αG (Fig. 5A). The ring of the ligand is buried inside the protein and the functional group protrudes out into the solvent (Fig. 5B). Multiple sequence alignment analysis with ConSurf^21^ shows that this site is highly conserved (Fig. 5B). The modeled ligand forms no hydrogen bonds with the protein, as judged by a cutoff distance of 3.2 Å, and appears to be stabilized mainly by nonpolar interactions. However, there are a few charged and polar groups near the ligand, which might provide some electrostatic stabilization. For example, Arg6 and a water molecule are within 4.6 Å and 3.3 Å of the amide, and Glu140 is 3.5 Å from the N heteroatom (Fig. 5A). Interestingly, the unknown ligand is not present in the trigonal structure in which the rigid body rotation of the C-terminal arm eliminates the binding pocket.

### Identification of potential ligand binding hot spots

**Figure 6:**
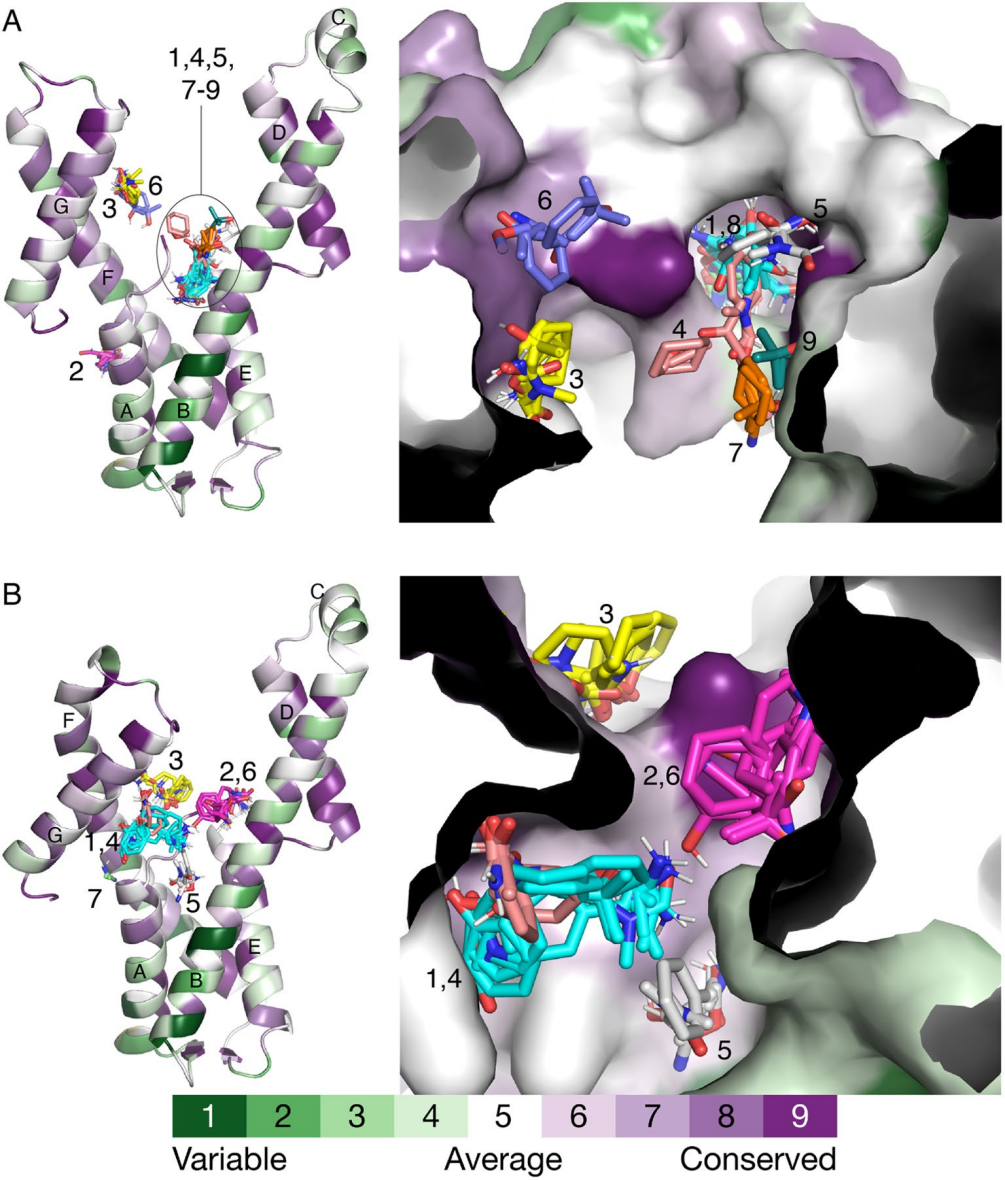
FTMap and ConSurf results. (**A**) Consensus clusters of the *P*3_2_21 structure. Clusters are labeled with numbers. Helices are labeled with letters. The protein is colored according to sequence conservation calculated with ConSurf. (**B**) Consensus clusters of the *C*222_1_ structure. Clusters are labeled with numbers. Helices are labeled with letters. The protein is colored according to sequence conservation calculated with ConSurf.

Docking of small organic molecules as implemented in FTMap was used to identify potential ligand binding hot spots^22^. Docking of 16 organic molecules against the *P*3_2_21 structure produced nine consensus clusters of poses (Fig. 6A). Six of the clusters are close together, forming a supercluster (clusters 1, 4, 5, 7, 8, 9). The supercluster maps out a potential binding site formed by αB and αE, as well as the N-terminus. This hot spot is notable for its deep pocket, which contains the largest consensus cluster (#1 in cyan in Fig. 6A). Two smaller clusters are in shallow depressions on the surface of the C-terminal arm (#3 and #6). Cluster 2 marks a pocket on the side of the 4-helix bundle. The supercluster, as well as clusters 3 and 6, occupy regions of higher-than-average sequence conservation, especially cluster 6 and the deep pocket containing cluster 1 (Fig. 6A, right).

Docking against the *C*222_1_ structure generated six consensus clusters located in the groove between the two helical arms (Fig. 6B). Clusters 1, 2, and 4–6 are close together, forming another supercluster. Cluster 1/4 is unique to the *C*222_1_ structure; apparently, the 25° rigid body rotation of the C-terminal arm opens this site. Cluster 7 is the smallest and corresponds to the nicotinamide site. FTMap docked one molecule each of acetonitrile and methylamine into the nicotinamide pocket. Clusters 2, 3, and 6 occupy regions of relatively high sequence conservation (Fig. 6B, right).

### Accuracy of the AlphaFold model of AaDUF507

**Figure 7:**
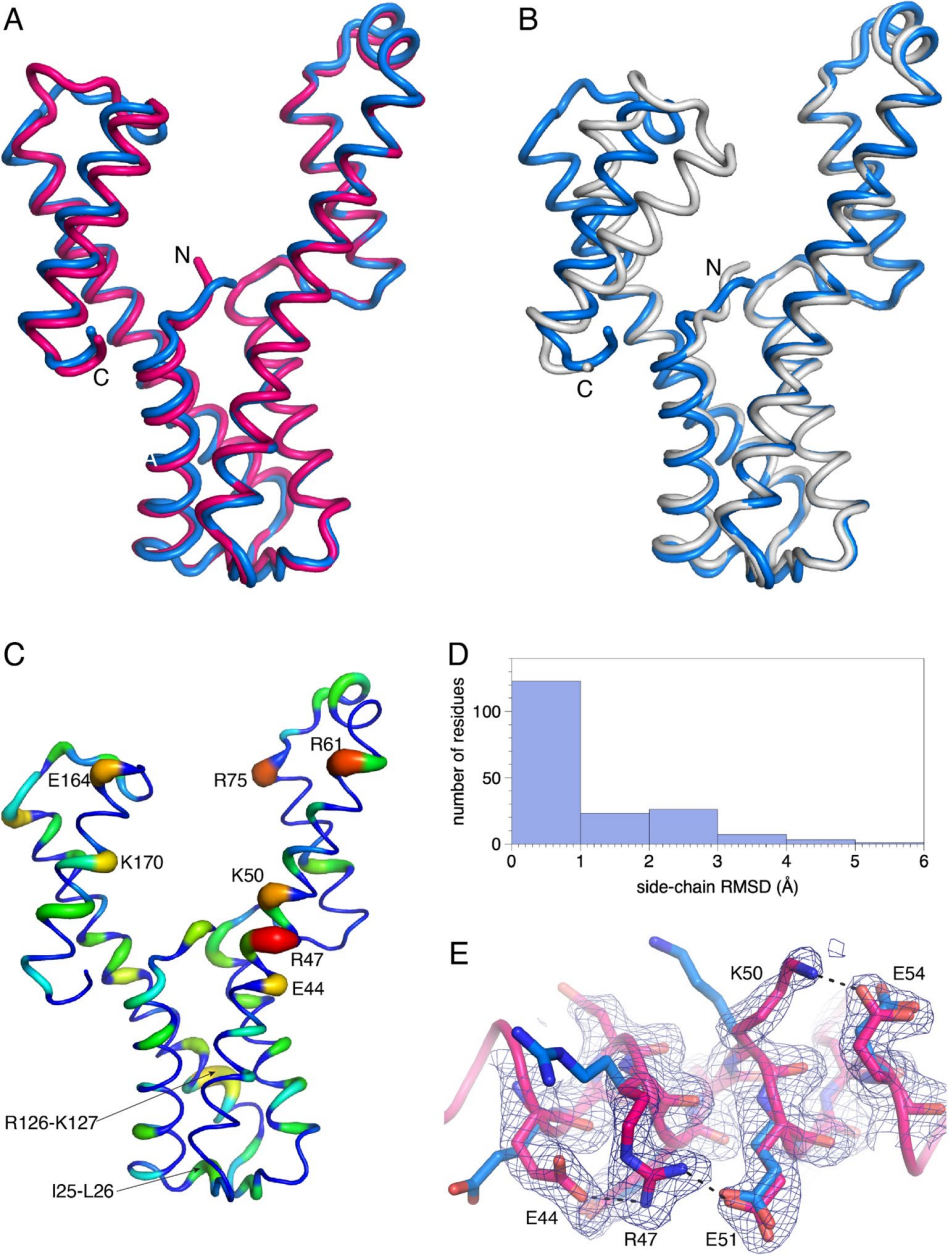
Comparison of the AlphaFold model to the crystal structures. (**A**) Superposition of the AlphaFold model (blue) and the *P*3_2_21 structure (hot pink). (**B**) Superposition of the AlphaFold model (blue) and the *C*222_1_ structure (white). (**C**) Sidechain RMSDs mapped onto the AlphaFold model. The width and color of the tube represents the sidechain RMSD between the AlphaFold model and the *P*3_2_21 structure. (**D**) Histogram of sidechain RMSDs (AlphaFold versus the *P*3_2_21 structure). (**E**) Example of sidechain conformational differences between the AlphaFold model (blue) and the *P*3_2_21 structure (pink). The mesh represents 2*F*_o_ − *F*_c_ density (1σ).

We used the crystal structures of AaDUF507 to assess the accuracy of the AlphaFold model. AlphaFold correctly predicted both the secondary and tertiary structure. The backbone Cα RMSD between the AlphaFold model and the *P*3_2_21 structure is only 1.2 Å (Fig. 7A). This value is impressive considering that the RMSD between the two crystal structures is twice as high at 2.4 Å. The RMSD between the AlphaFold model and the *C*222_1_ is considerably higher at 2.8 Å (Fig. 7B). The high deviation is due to a rotation of the C-terminal arm; compared to the *C*222_1_ structure, the C-terminal arm domain is rotated by 29° in the AlphaFold model.

The accuracy of the sidechains in the AlphaFold model was also assessed. This analysis was performed with the trigonal structure since it has the lower backbone RMSD to the AlphaFold model. AlphaFold predicted the sidechain conformations to within 1 Å RMSD of the crystal structure for 65% of the residues (Fig. 7D). Most of the sidechains with high RMSD (> 2 Å) are charged residues on the surface of the protein (Fig. 7C), and of these, many are involved in crystal contacts (e.g., Lys50, Arg61, Arg75, Arg126, Lys127, Glu164, and Lys170). An ion pair network on the surface of αB is one area where the AlphaFold model and crystal structure differ significantly (Fig. 7E). In both crystal structures, Arg47 ion pairs to Glu44 and Glu51; however, the former two sidechains have different conformations in the AlphaFold model and these ion pairs were not predicted.

## Discussion

Our results provide new information relevant to the classification of the DUF507 family. DUF507 proteins are found in bacteria and have two architectures. AaDUF507 represents the larger architecture, which consists of ~ 180 residues. The shorter architecture proteins have ~ 90 residues and are similar in sequence to the C-terminal half of AaDUF507. Presumably the structure of the shorter DUF507 proteins resembles the C-terminal half of AaDUF507. Thus, we suggest that the 3-helix motif found in the C-terminal half of AaDUF507 is the defining structural feature of the family, and that it is duplicated in the longer architecture DUF507 proteins, such as AaDUF507. The pseudo-twofold structural symmetry of AaDUF507 may have arisen through gene duplication and fusion, which has been proposed to be an origin of internal symmetric structure of monomeric proteins^23^. The structural symmetry also suggests that the shorter DUF507 proteins may dimerize via an intermolecular 4-helix bundle that mimics the intramolecular 4-helix bundle described here.

The structures may be useful for determining the function of DUF507 proteins. The electron density for one of the structures indicated an unknown small molecule, possibly nicotinamide, bound in a pocket of high sequence conservation. Unknown ligands are common in structures of proteins of unknown function. A survey of structures determined in the Protein Structure Initiative indicates that 5% of structures of proteins of unknown function have unknown ligands bound^24^. For example, a similar electron density feature was observed in another structure of a helical protein with unknown function^25^. In the structure of HP0721 from *Helicobacter pylori* (PDB 2XRH), the unknown ligand was modeled as nicotinic acid based on the strong interaction with an arginine sidechain, implying a carboxylate functional group. The lack of a positively charged sidechain or hydrogen bond donors close to the ligand functional group in our structure made nicotinamide seem more plausible than nicotinic acid. Also, similarly shaped electron density features representing an unknown ligand with the topology of a 6-membered ring with a sp^2^ functional group have been observed in structures deposited by the JCSG structural genomics project, including PDB entries 3GBH, 3NRB, 4J8P, and 2IG6.

Adventitious, unknown ligands in crystal structures can often aid in functional characterization^26^. The unknown ligand in AaDUF507 binds in the junction between the 4-helix bundle base and the C-terminal arm. The appearance of this pocket is correlated with the angle of the C-terminal domain. With the C-terminal domain rotated as in the *C*222_1_ structure, the arms of the Y are closer together and the pocket opens. Rotation of the C-terminal domain in the opposite direction, as in the *P*3_2_21 structure, opens the arms of the Y and closes the nicotinamide pocket. The disappearance of the nicotinamide pocket is accompanied by the appearance of a deep pocket near the N-terminal arm of the *P*3_2_21 structure, which was found by fragment mapping. These results suggest the possibility that the unknown ligand pocket is an allosteric site that tunes a larger functional ligand binding site between the arms.

Finally, AlphaFold correctly predicted both the secondary and tertiary structures of AaDUF507. Further, 65% of the sidechains were correctly predicted to within 1 Å RMSD of the crystal structure. The tertiary structure of the AlphaFold model is more similar to the *P*3_2_21 structure (RMSD of 1.2 Å) than the *C*222_1_ structure (RMSD of 2.8 Å), reflecting a difference in the angle of the C-terminal domain in the two crystal structures. Nevertheless, the AlphaFold model proved to be a suitable molecular replacement search model for the *C*222_1_ structure.

## Methods

### Protein expression and purification

A synthetic gene encoding DUF507 from Aquifex aeolicus (AaDUF507, UniProt O67633, 183 residues) with codons optimized for expression in *Escherichia coli* was synthesized and ligated into plasmid pET-24b( +) by GenScript. The expressed protein includes an N-terminal His-tag and TEVP cleavage site with the sequence MHHHHHHSSGVDLGTENLYFQ|S.

AaDUF507 was expressed in *E. coli* as follows. The plasmid was transformed into Invitrogen One-Shot BL21(DE3) competent cells. The transformed cells were then plated onto LB agar plates with 50 µg/mL kanamycin. 15 mL cultures were grown overnight on an orbital shaker at 195 rpm and 37 °C, and then used to inoculate 1 L cultures of TB media. The 1 L cultures were grown at 37 °C and 200 rpm until approximately 0.9 OD_600_ was obtained. The temperature was then reduced to 18 °C and protein expression was induced with 0.5 mM IPTG and continued overnight. The cells were collected by centrifugation at 4 °C for 30 min using a SLC6000 rotor at 5000 rpm. The resulting pellet was stored at -20 °C until ready for purification.

Expressed AaDUF507 was extracted from *E. coli* cells as follows. The frozen cells were thawed and resuspended in lysis buffer on ice. The lysis buffer contained 20 mM HEPES, 300 mM NaCl, 5% glycerol, and Pierce™ EDTA-free protease inhibitor tablets, 1 tablet per 50 mL of resuspended cells. The resuspended cells were lysed on ice using sonication. Sonication (Branson Sonifier 450 with duty cycle of 50% and Output control of 7) was applied in 2 min intervals (2 min on, 2 min rest) for a total of 14 min. The mixture was then centrifuged at 16,500 rpm using a SS-34 rotor (4 °C for 1 h) and the supernatant collected for chromatography steps.

AaDUF507 was purified as follows. The crude lysate from cell disruption was applied to a Cytiva 5 mL Histrap™ HP column (charged with Ni^2+^) at 2.5 mL/min using an Akta Start protein purification system. The column had been pre-equilibrated with buffer A consisting of 20 mM HEPES pH 7.5, 300 mM NaCl, 3% glycerol, and 30 mM imidazole. The resin was washed with 3–4 column volumes of buffer A at 1.5 mL/min. A linear elution gradient from 30 mM imidazole to 300 mM imidazole was applied over 50 column volumes. Fractions were analyzed with SDS-PAGE and pooled. The His-tag was removed by adding TEVP to the AaDUF507 sample and then dialyzing at room temperature overnight against 20 mM HEPES pH 7.5, 300 mM NaCl, 3% glycerol, and 1 mM TCEP. The next day, the sample was passed over the Histrap column and tag-free AaDUF507 was collected in the flow-through. The sample was then concentrated using an Amicon Ultra 15 10 k MWCO centrifugal concentrator to 13 mg/mL in preparation for size exclusion chromatography. Size exclusion chromatography was performed on an Akta Pure instrument with a HiLoad™ 16/600 Superdex™ 200 pg column. The column buffer contained 20 mM HEPES pH 7.5, 150 mM NaCl, and 3% glycerol. Fractions were pooled based on the chromatogram and concentrated to approximately 16 mg/mL. The concentration was estimated using absorbance at 280 nm assuming Abs 0.1% of 0.78 calculated with ProtParam^27^.

### Crystallization

Initial crystallization conditions were identified by screening the protein against several commercial crystal screens, including Hampton Crystal Screen HT and Molecular Dimensions BCS Eco Screen. The screens were performed in Swissci MRC 2 sitting drop plates set up with an Oryx-8 robot. The concentration of the protein stock solution was 16 mg/mL. The crystallization drop size consisted of 0.70 µL of protein and 0.30 µL of reservoir. Two crystal forms suitable for high resolution structure determination were obtained (centered orthorhombic and trigonal). The centered orthorhombic crystals were grown using a reservoir containing 0.01 M Co(II)Cl_2_ hexahydrate, 0.1 M sodium acetate pH 4.6, and 1.0 M 1,6-hexanediol, which was obtained after optimization of the hit from Crystal Screen HT condition E11. The trigonal crystal used for structure determination was harvested directly out of the crystal screen (Molecular Dimensions BCS Eco Screen kit well D8), which had a reservoir solution of 0.1 M MES monohydrate pH 6.5 and 12% w/v PEG 20,000. The centered orthorhombic crystals were cryoprotected in 35% ethylene glycol. The trigonal crystals were cryoprotected in 25% PEG200.

### X-ray diffraction data collection, phasing and refinement

Shutterless X-ray diffraction data were collected at beamline 4.2.2 of the Advanced Light Source using a Taurus-1 CMOS detector. The data were integrated and scaled with XDS^28^. Intensities were converted to amplitudes with Aimless^29^. Analysis of the data sets with Phenix^30^ Xtriage indicated a pathology of “moderate anisotropy” for the centered orthorhombic data and no obvious pathologies for the trigonal data set. Data processing statistics are listed in Table 1.

The phase problem was solved using molecular replacement as implemented in MOLREP^31^ and Phaser^32^. The centered orthorhombic structure was solved first. Because AaDUF507 has no detectable sequence similarity to any protein in the PDB, an AlphaFold model obtained from UniProt was used as the search model^33^. The cross rotation function calculated in MOLREP showed a single prominent peak, consistent with one molecule in the asymmetric unit. Molecular replacement calculations in Phaser produced a solution with one molecule in the asymmetric unit of space group *C*222_1_, implying a solvent content of 59% and *V*_M_ of 3.0 Å^3^/Da^34^. Visual inspection of the model and map from Phaser showed no severe intermolecular steric clashes and strong electron density throughout the protein chain except for residues 146–183, which correspond to the C-terminal arm. Attempts to obtain a solution with two molecules in the asymmetric unit were unsuccessful (returned the one-molecule solution).

Automated model building and refinement in Phenix^35–37^ were used to validate the molecular replacement solution and complete the model. The model from molecular replacement was input to simulated annealing refinement in Phenix, which resulted in *R*_work_ of 0.37 and *R*_free_ of 0.43. The map from simulated annealing refinement was then input to automated building in Phenix Autobuild. To reduce model bias, the model from refinement was not used in automated building. The model from the first round of automated building included residues 7–182 assigned to sequence (out of 183 total) with *R*_work_ of 0.25 and *R*_free_ of 0.30. The model from automated building was improved through manual building in Coot^38,39^ and refinement in Phenix. A few more iterations of automated building followed by manual building and crystallographic refinement were performed until a nearly complete structure was obtained. Then several cycles of manual building followed by refinement in Phenix were performed to complete the structure. The final *C*222_1_ structure includes residues 1–183, 91 water molecules, and one molecule of 1,6-hexanediol. Structure validation was performed using MolProbity and the wwPDB validation server^40,41^. Refinement statistics are listed in Table 1.

The final *C*222_1_ structure served as the search model to solve the trigonal structure. The search model was prepared by removing solvent and trimming sidechains to the Cγ atoms. Molecular replacement calculations in Phaser returned a solution having one molecule in the asymmetric unit of space group *P*3_2_21, implying 60% solvent and *V*_M_ of 3.1 Å^3^/Da. The structure was completed using the strategy outlined for the *C*222_1_ structure. The final structure includes residues 1–183, 55 water molecules, a sodium ion, and two PEG fragments. Refinement statistics are listed in Table 1. Coordinates and structure factor amplitudes for both structures have been deposited in the PDB under the accession codes 8T8K and 8T8L.

### Circular dichroism

The secondary structure of AaDUF507 was studied with CD. The protein sample was exchanged into a buffer containing 10 mM potassium phosphate at pH 7.5 (CD buffer) using an Amicon Ultra 4 10,000 NMWL centrifugal concentrator, adding CD buffer to sample and spinning (repeated 3 times). The sample was then diluted with CD buffer to 0.47 mg/mL. The CD spectrum was recorded on a Chirascan V100 instrument at 24 °C using 1 mm pathlength matching cuvettes.

### Small-angle X-ray scattering (SAXS)

In preparation for SAXS, a sample of purified AaDUF507 was dialyzed into 20 mM HEPES pH 7.2 and 400 mM NaCl using a Slide-A-Lyzer mini dialysis 10,000 MWCO unit. The sample was concentrated to 16 mg/mL using an Amicon 4 centrifugal concentrator. Samples were diluted to concentrations of 1–16 mg/mL and pipetted into a 96-well flat bottom plate and shipped at 4 °C to ALS beamline 12.3.1. SAXS buffer and concentrator flow-through were included in the plate bracketing the protein samples for buffer subtraction.

SAXS data collection was performed at 20 °C using a Pilatus detector at beamline 12.3.1 of the Advanced Light Source through the SIBYLS Mail-in HT-SAX program^42^. Data were collected in shutterless mode with a total exposure of 10 s, framed every 0.3 s. The sample-to-detector distance was 2 m and the wavelength was 1.234 Å. Buffer-subtracted frames were averaged using SAXS FrameSlice^43^. For the data reported here (1 mg/mL), the following frames were used in averaging: Guinier region, frames 1–2; Porod region, frames 1–15; wide region, frames 1–25. Primus 3.2.1 was used for Guinier analysis and calculations of the distance distribution function^44^. The molecular weight was estimated using the methods of volume of correlation^45^, SAXS MoW^46^, and Bayesian inference^47^ as implemented in the Primus 3.2.1 Molecular Weight utility. FoXS was used to calculate scattering curves from the crystal structure^14,15^. The SAXS data, *P*(r) curve, and best fit model have been deposited in the SASBDB under accession code SASDSX6.

### Computational analysis

Domain rotations were analyzed with DynDom^11^. Structural queries of the PDB were performed with TopSearch^17^, DALI^18^, and PDBeFold^12^. Sequence alignments were performed with NCBI BLASTP^13^ and Clustal Omega^48^, and visualized with ESPript 3.0^49^. RMSD calculations were done with CNS^10^. The sidechain RMSD for each residue was calculated after first superimposing the backbone of the residue, and care was taken to exclude spurious differences that arise for amino acids with groups exhibiting mirror symmetry (i.e., rings of Phe and Tyr; carboxylates of Asp and Glu). The ConSurf server^21^ was used to calculate multiple sequence alignments and map the results onto the structure.

## Data Availability

The coordinates and structure factor amplitudes of AaDUF507 are available from the Protein Data Bank under access codes 8T8K and 8T8L. The diffraction images for both structures have been deposited at proteindiffraction.org.^50^ The SAXS data, *P*(r) curve, and best fit model have been deposited in the SASBDB under accession code SASDSX6.

## Abbreviations

AaDUF507: DUF507-containing protein from *Aquifex aeolicus*
CD: Circular dichroism
DUF: Domain of unknown function
SAXS: Small-angle X-ray scattering
